# Magnet-Facilitated Selection of Electrogenic Bacteria from Marine Sediment

**DOI:** 10.1155/2015/582471

**Published:** 2015-10-04

**Authors:** Larisa Kiseleva, Justina Briliute, Irina V. Khilyas, David J. W. Simpson, Viacheslav Fedorovich, M. Cohen, Igor Goryanin

**Affiliations:** ^1^Biological Systems Unit, Okinawa Institute of Science and Technology, 1919-1 Tancha, Onna-son, Okinawa 904-045, Japan; ^2^Institute of Fundamental Medicine and Biology, Kazan (Volga Region) Federal University, Ulitsa, Kremlyovskaya 18, Kazan, Republic of Tatarstan 420008, Russia; ^3^School of Informatics, University of Edinburgh, 10 Crichton Street, Edinburgh EH8 9AB, UK

## Abstract

Some bacteria can carry out anaerobic respiration by depositing electrons on external materials, such as electrodes, thereby creating an electrical current. Into the anode chamber of microbial fuel cells (MFCs) having abiotic air-cathodes we inoculated microorganisms cultured from a magnetic particle-enriched portion of a marine tidal sediment, reasoning that since some external electron acceptors are ferromagnetic, electrogenic bacteria should be found in their vicinity. Two MFCs, one inoculated with a mixed bacterial culture and the other with an axenic culture of a helical bacterium isolated from the magnetic particle enrichment, termed strain HJ, were operated for 65 d. Both MFCs produced power, with production from the mixed culture MFC exceeding that of strain HJ. Strain HJ was identified as a *Thalassospira* sp. by transmission electron microscopic analysis and 16S rRNA gene comparisons. An MFC inoculated with strain HJ and operated in open circuit produced 47% and 57% of the maximal power produced from MFCs inoculated with the known electrogen *Geobacter daltonii* and the magnetotactic bacterium *Desulfamplus magnetomortis*, respectively. Further investigation will be needed to determine whether bacterial populations associated with magnetic particles within marine sediments are enriched for electrogens.

## 1. Introduction

As a group, bacteria obtain electrons for respiratory metabolism from a vast range of sources and, likewise, deliver these electrons to an equally impressive array of acceptor molecules. A growing list of bacteria have been found to be electrogenic, that is, capable of reducing external solid electron acceptors. From a biotechnological standpoint electrogenic bacteria are of interest because they can efficiently oxidize compounds within the anaerobic environment of microbial fuel cells (MFCs) while creating an electrical current [1].

Among the electrogenic bacteria a variety of mechanisms are employed for reducing external electron acceptors [2]. Two types of “nanowires” have been identified by which cells can deliver electrons from cell membrane respiratory proteins: type IV pili, found in* Geobacter* spp., and cytochrome-containing outer membrane extensions found in* Shewanella* spp. [3, 4]. Bacteria may stably associate with a solid electron acceptor surface as attached biofilms [5] or transiently by electrokinesis, a dynamic process in which bacteria cycle between depositing electrons and swimming in the vicinity of the acceptor [6]. Some bacteria are also capable of transferring electrons to external solid electron acceptors via diffusible electron carrying shuttle molecules, some of which can be exchanged between species [7]. Other unidentified mechanisms for external electron delivery almost certainly exist. Development of means to screen for and isolate electrogens would aid in the discovery of such mechanisms.

Conductive minerals (e.g., magnetite Fe_3_O_4_) confer centimeter-long conductivity to anaerobic marine sediments [8], promoting the activity of electrogens in these environments, and enabling applications such as powering of remote devices and reductive dechlorination of contaminants [9]. Magnetite can facilitate electron transfer from bacteria to external receptors [10], including transfer to nitrate-reducing bacteria [11], and under acidic conditions magnetite it can be an external electron acceptor [12]. Recently, it was demonstrated that magnetite, owing to its mixed valency, can behave as a battery, being oxidized by phototrophic bacteria in the light and reduced by electrogenic bacteria in the dark [13]. We reasoned, therefore, that electrogenic bacteria may be found in preferential association with magnetic particles of marine sediments. Here we describe the isolation and partial characterization of an electrogenic bacterium* Thalassospira* sp. strain HJ from a magnetic particle-enriched portion of a marine tidal sediment.

## 2. Materials and Methods

### 2.1. Sediment Sampling and Preparation

Sandy sediment from Kaichu-Doro Beach (26° 19′ 56.1′′ N, 127° 54′ 0′′ E; October, 2013) Okinawa Japan, was sampled to 25 cm depth and placed into 500 mL bottles to approximately half capacity, filling the remainder of the bottle with seawater. At the laboratory, the sample was vigorously mixed and allowed to settle with a magnet pressed against the outside of the container positioned above the height of the sediment surface before mixing. After the sediment had settled, 4 mL of liquid and adherent magnetic particles from the region closest to the magnet was sampled with a sterile Pasteur pipette. The “capillary racetrack method” was then used to enrich for magnet-associated bacteria from the sample [14]. Fluid from the capillary was inoculated into a test tube containing 10 mL culture medium (Difco Marine Broth 2216) supplemented with 50 mg L^−1^ FeCl_3_ (Marine-Fe broth) and grown overnight at 23°C without shaking. A sample was streaked to Marine-Fe Agar, incubated for 2 d, and a resulting colony of a spiral bacterium (termed strain HJ) was subcultured into Marine-Fe broth. 50 mL subcultures of both the original capillary racetrack-derived mixed community and strain HJ were poured into separate MFCs that were topped off with ~125 mL 0.1 M sodium/potassium phosphate buffer (pH 5.9) and 1 mL 2% sodium acetate.

### 2.2. Operation of Sediment-Derived Culture-Inoculated Microbial Fuel Cells

Two single-chamber, air-cathode MFCs (*H*, 10 cm; *L*, 12 cm; *W*, 10 cm) were prepared to have internal working volume of 175 mL. The internal MFC chamber contained two anodes (approximately 6 × 8 cm), suspended 2-3 mm off the bottom of the chamber, composed of a layer of 0.4 mm proprietary conductive carbon cloth to which 2 mm average size activated carbon granules were bound with conducted glue to provide more surface area. The granules had been prepared from birch precursor and pretreated with a neutral red catalyst to facilitate electron transfer. The two cathodes (6 × 8 cm) were graphite plates (3 mm thick; 60% porosity) sprayed on the liquid-facing side with an aqueous 5% Fumion membrane polymer (FuMA-Tech, Bietigheim-Bissingen, Germany) while activated carbon granules (treated with iron(II) phthalocyanine) were mechanically pressed to the air-facing side using netting frame. Unit cathode section (including membrane) was cleaned by immersion in concentrated HCl before module assembly for 20 min to remove any organics and was then soaked in sterile distilled water, with water changes every 1 h until the pH was neutralized. The reactor cell PVC surfaces were cleaned by washing with soap and water, drying, and then wiping down with acetone solvent. Anode and internal reactor areas were sprayed with 70% ethanol solution just prior to final assembly. The cathode extended into a bath containing an electrolyte solution maintained at pH 2 with regular additions of 1 N HCl to provide a source of protons for the abiotic reduction of oxygen to water. The MFCs were maintained on a 24 h open circuit/24 h closed circuit cycle, using an external resistance of 40 Ω. The anode and cathode electrodes were connected with a multichannel logger (Graphtec midi LOGGER GL820, Japan) for daily voltage measurements. The corresponding electric current was calculated using Ohm's law (*V* = *IR*). Power density was obtained according to the equation *P* = *IV*/*A*, where *I* is the current, *V* is the voltage, and *A* is the projected surface area of the cathode.

Once weekly, after removal of 1 mL mixed contents for chemical oxygen demand (COD) analysis, the MFCs were fed with approximately 5 mL phosphate buffer containing 1 g L^−1^ sodium acetate when COD analysis indicated substrate depletion. The extra volume of the feed was needed to replace losses due to evaporation through the cathode membrane. Upon completion of the monitoring period on the 65th day of operation, due to declining power production, the MFCs were disassembled. Anode material was sampled and bacteria were isolated from the strain HJ-inoculated MFC to confirm the presence of strain HJ.

### 2.3. Bacterial Isolation and Initial Characterization

DNA from strain HJ was isolated and subject to genomic sequencing [15]. Phylogenetic analyses of the 16S rRNA gene (GenBank accession number KP704219) were performed using the Phylogeny.fr platform [16]. Minimum and maximum temperatures for growth were determined by culturing on Marine Agar plates. Catalase and oxidase activities were tested and Gram staining was carried out using standard microbiological methods.

### 2.4. Transmission Electron Microscopy

Transmission electron microscope (TEM) imaging was performed on a JEOL JEM-1230R Electron Microscope at an accelerating voltage of 100 KV. Strain HJ cells were prepared from cultures grown at room temperature (23°C) in 50 mL of Marine-Fe broth to an OD_600_ of 1.0. The pelleted cells were fixed with 1% osmium tetroxide in 0.1 M cacodylate buffer (pH = 7.2) for 30 min and washed with water three times for 5 min. A drop of fixed bacterial cells was deposited onto a carbon-coated HF34 200 mesh copper grid, washed once with distilled water, and stained for 3 to 5 sec with 1% uranyl acetate.

### 2.5. Inoculation and Monitoring of Microbial Fuel Cells Inoculated with Axenic Bacterial Cultures

A micro-MFC array was developed from two parts of Plexiglas with three 4 cm microchambers to test for current generation by three axenic bacterial cultures. Each chamber consisted of an anode and a cathode compartment (8 mm deep) separated by a cation-exchange membrane Nafion 117 (19.6 cm^2^; DuPont Co., Delaware USA). The anode and cathode electrodes (3 mm thick graphite plates; 45–50% porosity, Xinghe County Muzi Carbon Co., Ltd, China) were connected with a multichannel logger (Graphtec midi LOGGER GL820, Japan) for daily voltage measurements. 1 mm thick rubber gaskets were used for sealing between the anode and cathode compartments. A titanium screw held the electrode against the membrane and acted as electrical connector. Each micro-MFC chamber was equipped with a 2.5 ID mm polyurethane inlet tube (FESTO, Germany) on the bottom for medium and electrolyte feeding and one on the top for biogas output and replacement of exhausted electrolyte. For disinfection the plexiglas microchambers were soaked in a 10% bleach solution, rinsed with deionized water, and then exposed to UV light for 12 h. Graphite plates were washed with 100% isopropanol in an ultrasonic bath and then heated in an oven for 2 h at 200 C. Micro-MFCs were operated in the open circuit mode and current measurements were performed every 24 hours starting from time of inoculation. 50 mM iron(II) phthalocyanine, without N_2_ sparging, was used as the cathode electrolyte solution.

Axenic bacterial cultures (*Thalassospira* sp. HJ, the known electrogen* Geobacter daltonii*, and the magnetotactic* Desulfamplus magnetomortis*) were pregrown with shaking at 37°C for 12 h in 5 mL of modified PBS medium containing (per liter) 4.58 g Na_2_HPO_4_, 2.45 g NaH_2_PO_4_·H_2_O, 0.31 g NH_4_Cl, 0.13 g KCl, 5 g glucose, 5 g yeast extract, and 5 g peptone. Bacterial cells were harvested, washed with 16 mM phosphate buffer (pH 7.0), and resuspended into 50 mL fresh modified PBS medium. The initial cell concentration was adjusted to an optical density at 600 nm of 0.3 as measured by a Spectronic GENESYS 5 spectrophotometer (Milton Roy Company, Rochester, NY, USA) with filtered cell-free culture medium as reference.

The micro-MFCs were inoculated initially with 15 mL medium containing bacterial cells (exceeding the ~10 mL volume of the anode chamber since the porous graphite absorbed some liquid). Bacteria-free modified PBS medium containing 0.1% NaN_3_ was used for control experiments. All experiments were set up in two technical replications and two biological replications. The axenicity of the anolytes for the strain HJ-containing MFCs was determined at the end of the experiments by plating serial dilutions to Marine Agar medium. Experiments were performed at room temperature.

## 3. Results and Discussion

**Figure 1:**
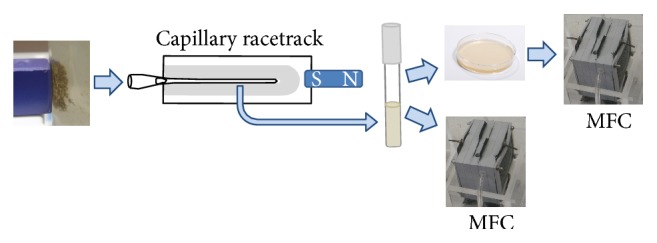
A flow scheme indicating the procedure for enrichment of magnetic particles from sediment and inoculation of marine broth medium and microbial fuel cells.

**Figure 2:**
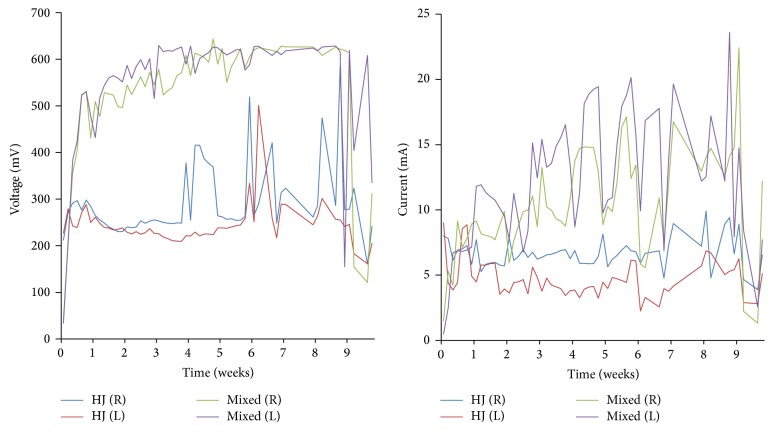
Electrical output from microbial fuel cells inoculated with cultures derived from tidal beach sediment obtained from Kaichu-Doro Beach, Okinawa, Japan. Total anode surface area, 151 cm^2^ (75.5 cm^2^ per anode); 175 mL volume. Plotted lines indicate activity from the mixed culture-inoculated MFC and the strain HJ-inoculated MFC; R, right, and L, left anode-cathode couple.

To obtain magnetic particle-associated bacteria, some of which should be electrogenic [12], we subjected tidal beach sediment to the enrichment procedure depicted in Figure 1. One MFC was inoculated with a mixed culture derived from the magnetic particle-enriched sediment and the other with an axenic culture of a helical bacterium, strain HJ, isolated from the enriched sediment. Both MFCs were found to produce power, with the current and voltage generated from the mixed culture MFC being substantially higher (Figure 2). The comparatively low power density observed of strain HJ is typical of axenic bacterial cultures relative to that of the communities from which they are derived [5]. At the end of the experiment strain HJ was reisolated from the MFC to confirm its retention.

**Figure 3:**
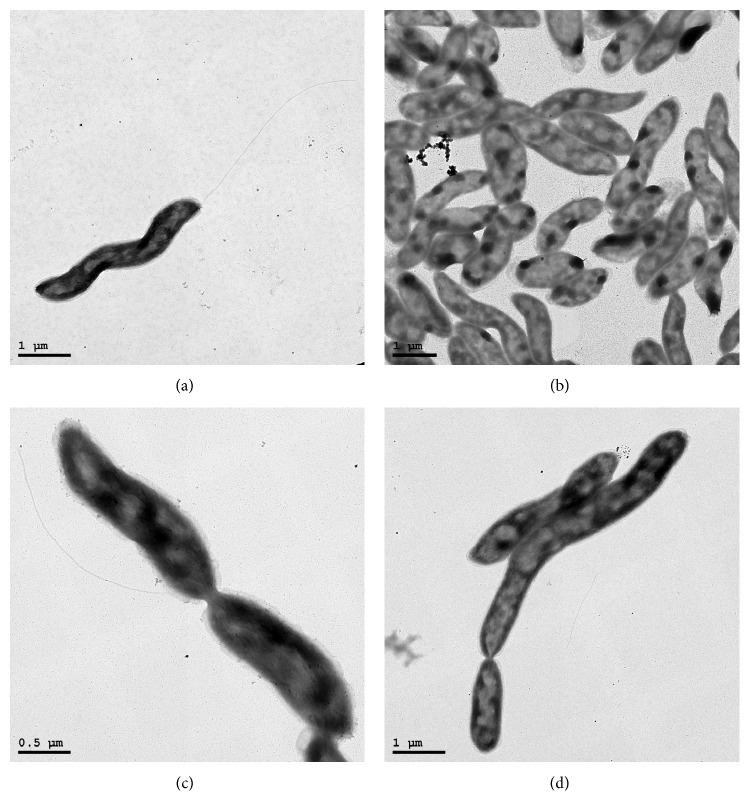
Transmission electron micrographs of* Thalassospira* sp. strain HJ showing various features. (a) A single polar flagellum; (b) intracellular electron-dense regions; (c) symmetric cell division; and (d) asymmetric cell division. Representative images are shown.

**Figure 4:**
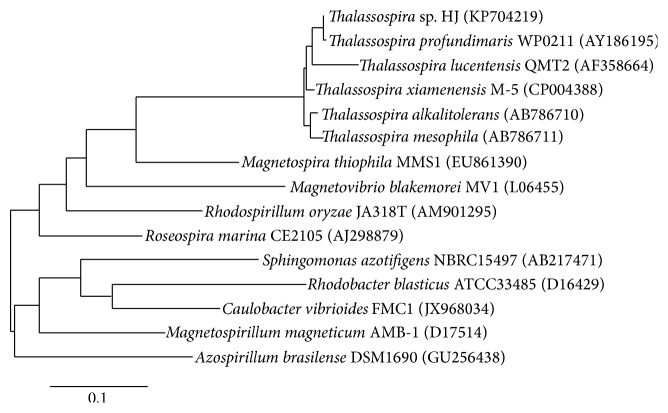
Phylogenetic tree of strain HJ and selected alphaproteobacteria based on 16S rRNA gene sequences. The tree was constructed from sequences aligned by MUSCLE using maximum-likelihood method of PhyML 3.0 [16, 21]. The scale bar reflects evolutionary distance, measured in units of substitution per nucleotide site.

Strain HJ was identified by means of microscopic analysis (Figure 3) and 16S rRNA gene sequence comparisons (Figure 4) to be of the genus* Thalassospira*. TEM images show cells of 0.5–0.7 *μ*m width and 1.5–5.7 *μ*m length having single polar flagella typical of the genus* Thalassospira* [17], with evidence of both symmetric and asymmetric cell division (Figure 3). One or more electron-dense staining regions can be seen within most cells (Figure 3). Cells were motile, stained Gram negative, and tested positive for catalase and oxidase activity. Growth occurred on Marine Agar at temperatures ranging from 12°C to 39°C and pH values ranging from 5.5 to 10.5. The closest matching 16S rRNA gene sequence to a named species within the GenBank database was that of* Thalassospira profundimaris* strain WP0211 (1449/1453, 99.7% identity), which notably differs from strain HJ in its lack of flagella [18].

**Figure 5:**
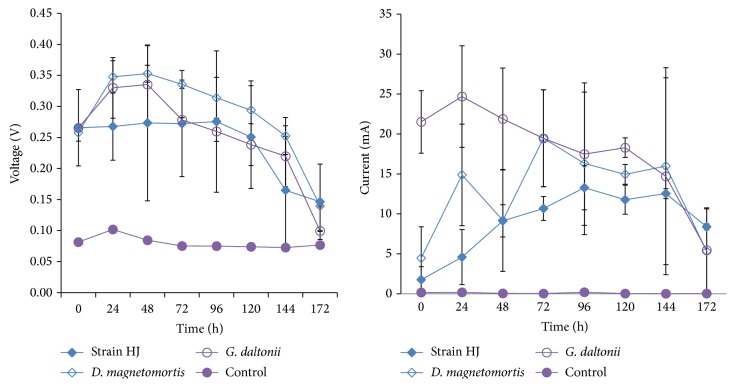
Voltage and current readings (±range; *n* = 2) from microbial fuel cells inoculated with axenic cultures of* Thalassospira* sp. strain HJ,* Geobacter daltonii*, and* Desulfamplus magnetomortis* or medium only with 1 g L^−1^ sodium azide (control). Anode surface area, 19.6 cm^2^; 10 mL volume.

Micro-MFC chambers inoculated with axenic cultures of* Thalassospira* sp. strain HJ and, for comparison,* Geobacter daltonii* and* Desulfamplus magnetomortis* showed electrical current production immediately after inoculation but differed in the intensity and shape of the subsequent production curve (Figure 5).* Thalassospira* sp. strain HJ and* D. magnetomortis* displayed gradual increases in current after initiation whereas current production from* G. daltonii* decreased with time. After 172 h, the current for all axenic cultures decreased to 5–8 mA, which was attributed with C-source depletion. Voltage drop was observed after 144 h from the strain HJ-inoculated micro-MFC, whereas* G. daltonii* and* D. magnetomortis* MFCs demonstrated voltage loss after 48 h. As a control, to determine the contribution of growth medium components to current and voltage production, a micro-MFC was filled with sterile medium. Although there was no observable current generation background voltage was observed (Figure 5). The maximal single-value power readings from the micro-MFCs were of 2.6 W m^−2^ (at 96 h) for* Thalassospira* sp. strain HJ, 5.5 W m^−2^ (at 144 h) for* D. magnetomortis*, and 4.6 W m^−2^ (at 24 h) for* G. daltonii*.

*Geobacter* is a well-characterized genus of electrogenic bacteria and although we are not aware of prior reports of electrogenicity by the magnetotactic bacterium* D*.* magnetomortis* members of sulfate-reducing members of the Deltaproteobacteria, to which* D*.* magnetomortis* belongs, were found to display transcriptomic responses to changes in MFC electrode potential indicative of electrogenic behavior [19]. We hypothesize that magnetosomes could confer a selective benefit by enabling bacteria to hone in on ferromagnetic external electron acceptors. A wider survey of magnetotactic bacteria for electrogenicity would be warranted.

Recently, two marine sediment-derived* Thalassospira* sp. isolates were reported to exhibit electrotrophic behavior, accepting electrons from insoluble sulfur [20] but the capacity of these strains to transfer electrons to an anode as we have found here of* Thalassospira* sp. strain HJ was not examined. Examination of the strain HJ genome does not reveal any homologs for the pili or outer membrane extension-type external electron transfer systems found in* Geobacter* or* Shewanella* species [15], and microscopic imaging of anodes from strain HJ-colonized MFCs implies that biofilm formation is not necessary for electrogenicity (data not shown). Thus, we hypothesize that external electron transfer occurs via an electron shuttle-type mechanism.

## 4. Conclusions

A microbial community derived from magnetic particle-enriched marine sediment displayed electrogenic behavior and yielded an electrogenic bacterium identified to be a* Thalassospira* species. This is the first report of electrogenic behavior within the genus* Thalassospira.* A more extensive study would be needed to determine whether the proportion of electrogenic bacteria obtained from this magnetic particle enrichment procedure exceeds that found in the environment. With their diurnal patterns of flooding and diversity of mineral components tidal sediments should be rich environments to bioprospect for electrogenic bacteria.
